# Pulmonary Infectious Complications in Children with Hematologic Malignancies and Chemotherapy-Induced Neutropenia

**DOI:** 10.3390/diseases8030032

**Published:** 2020-08-19

**Authors:** Aikaterini Voulgaridou, Kleoniki I. Athanasiadou, Eftychia Athanasiadou, Emmanuel Roilides, Evgenia Papakonstantinou

**Affiliations:** 1 Department of Pediatrics, General Hospital of Kavala, Agios Syllas, GR-655 00 Kavala, Greece; 2 School of Medicine, Aristotle University of Thessaloniki, University Campus, GR-541 24 Thessaloniki, Greece; kleoatha@auth.gr; 3 1st Department of Internal Medicine, 424 General Military Hospital, Ring Road, Efkarpia, GR-564 29 Thessaloniki, Greece; eftychiaath1@gmail.com; 4 Infectious Diseases Unit, 3rd Department of Pediatrics, Hippokration Hospital, Aristotle University School of Medicine, Konstantinoupoleos 49, GR-546 42 Thessaloniki, Greece; roilides@gmail.com; 5 Pediatric Oncology Department, Hippokration Hospital, Konstantinoupoleos 49, GR-546 42 Thessaloniki, Greece; eugepapa@yahoo.gr

**Keywords:** cancer, children, febrile neutropenia, infection, respiratory tract, chemotherapy

## Abstract

Infections frequently complicate the treatment course in children with hematologic malignancies undergoing chemotherapy. Febrile neutropenia (FN) remains a major cause of hospital admissions in this population, and respiratory tract is often proven to be the site of infection even without respiratory signs and symptoms. Clinical presentation may be subtle due to impaired inflammatory response. Common respiratory viruses and bacteria are widely identified in these patients, while fungi and, less commonly, bacteria are the causative agents in more severe cases. A detailed history, thorough clinical and basic laboratory examination along with a chest radiograph are the first steps in the evaluation of a child presenting signs of a pulmonary infection. After stratifying patient’s risk, prompt initiation of the appropriate empirical antimicrobial treatment is crucial and efficient for the majority of the patients. High-risk children should be treated with an intravenous antipseudomonal beta lactam agent, unless there is suspicion of multi-drug resistance when an antibiotic combination should be used. In unresponsive cases, more invasive procedures, including bronchoalveolar lavage (BAL), computed tomography (CT)-guided fine-needle aspiration or open lung biopsy (OLB), are recommended. Overall mortality rate can reach 20% with higher rates seen in cases unresponsive to initial therapy and those under mechanical ventilation.

## 1. Introduction

Hematologic malignancies account for approximately 40% of all childhood malignancies [1]. Although the vast majority of children with blood cancer succeed in long-term survival, there are some factors associated with significant morbidity and mortality. These factors are the nature of the disease and infiltrative bone marrow insufficiency, along with the intensity of the treatment protocols including chemotherapeutic drugs, use of corticosteroids, central lines or hematopoietic cell transplantation. Infectious complications still remain a major challenge during the course of childhood leukemia [2,3]. Nowadays, there have been great advances in stratifying children with febrile neutropenia (FN), in order to correspond to their risk of serious infection. FN is defined by the National Institute for Health and Care Excellence (NICE) as Absolute Neutrophilic Count (ANC) < 500/mm^3^ and fever as single temperature ≥ 38.5 °C or as ≥38 °C sustained for >1 h or occurring twice within a 24 h period [4]. FN-related infections, while they can occur at any point during treatment for acute lymphoblastic leukemia, are more frequently expected in the induction phase [5,6]. The respiratory tract of immunocompromised patients becomes a vulnerable and, therefore, an alluring target for microorganisms, pathogenic or opportunistic, as it is directly connected with the environment. In children with febrile neutropenia, respiratory infections can rapidly disseminate, leading to respiratory distress and pulmonary compromise [7,8]. For this reason, prompt diagnosis and management are of great importance, in order to reduce mortality rates [7,8]. We conducted this narrative review of the literature, using MEDLINE database, to present a holistic approach regarding the management of these cases and address the various challenges attending physicians are facing, in pediatric hematology-oncology units.

## 2. Discussion

### 2.1. Initial Presentation of a Respiratory Infection in a Child with Cancer

The clinical presentation of respiratory infections may vary widely. Respiratory viruses, which remain the most significant cause of infection, can present with a wide spectrum of manifestations mainly affecting, without being limited to, the respiratory tract. Clinical presentation ranges from mild, common cold-like symptoms to more severe lower respiratory tract symptoms requiring inpatient treatment and mechanical support [9]. Moreover, children with hematologic malignancies often have impaired immune response, which may mask clinical and laboratory findings of infections, due to attenuated inflammatory process. As a result, pneumonia may present only subacute fever, while respiratory symptoms, such as cough, dyspnea or pleuritic chest pain may be absent [10,11].

### 2.2. Basic Evaluation

The first step in the evaluation of a child with febrile chemotherapy-induced neutropenia is to take a detailed history including the dose of intensive chemotherapy treatment, the use of aggressive therapeutic modalities and recent environmental exposures and vaccinations and perform a meticulous physical examination [10]. Signs of respiratory infection may include crackles on auscultation, hypoxia and increased respiratory rate or strenuous breathing. Severe pneumonia can cause respiratory failure, septic shock, sepsis and multiple organ dysfunction.

Laboratory examinations should follow, according to recent guidelines for the management of FN in children with blood cancer, including complete blood count, electrolytes, measurement of C-reactive protein (CRP), procalcitonin (PCT), d-dimer and renal and liver function tests, along with blood cultures taken from each lumen of the central venous catheter (CVC) [12]. Additionally, blood cultures can be taken also from peripheral vein during the first episode of fever, in order to improve detection of the causative infectious agent.

In addition, the estimation of the Sequential Organ Failure Assessment (SOFA) score, developed by the European Society of the Intensive Care Medicine (ESICM), is recommended [13]. When respiratory signs and symptoms are present, studies such as culture and/or molecular analysis of nasopharyngeal swab or wash [14] together with chest x-ray (CXR) should be added to the management [12,15].

Regarding CXR, a cohort study of 88 patients with 170 episodes of FN concluded that the use of the chest radiograph at the time of initial evaluation of children with FN, when no respiratory symptoms were present, did not add any useful information, although it is a well-established step in adult management [15]. Importantly, in children with respiratory symptoms, negative CXR results should be interpreted with caution as infiltrate appearance may be delayed until neutrophil count normalization [10]. CXR is recommended in cases of persisting fever or in fever with a rising number of white blood cells and neutrophils. Bacterial infections cause non-specific radiological findings, usually alveolar consolidation with air bronchogram. Viral lung infections are presented as ground glass or reticular opacities. Fungi and especially *Aspergillus* spp. are revealed as nodular lesions surrounded by a “ground glass” halo or “meniscus” sign but other non-specific opacities may also be apparent. Moreover, it has to be emphasized that computed tomography (CT) scanning should be performed in any cases of prolonged FN lacking an established diagnosis.

The diagnosis of the viral infections is confirmed with viral culture, direct fluorescent antibody immunoassay or most frequently by multiplex polymerase chain reaction (PCR).

The diagnosis of mycoses depends on microscopy, culture in Sabourand agar, detection of galactomannan and PCR. Assay of 1,3-β-d-glucan, a cell wall component of most fungi, is positive in *Candida* spp., *Aspergillus* spp., *Fusarium* spp., *Acremonium* spp. and *Pneumocystis jirovecii* and negative in *Cryptococcus* spp., and mucormycosis. Testing for biomarkers of mycoses, like mannan antigen for *Candida* spp. and galactomannan for *Aspergillus* spp. (sensitivity 80–100%, specificity 90–100%), is common in everyday practice. Galactomannan is a cell wall component of growing hyphae and can be detected in serum, urine or bronchoalveolar lavage (BAL) samples [16].

### 2.3. Invasive Approaches

Deploying the results obtained from the previous testing, it is possible to tailor the most suitable combination of antimicrobial drugs. However, in spite of broad microbiological testing for bacterial, fungal and viral infections, most cases of FN infections yield no microbiological diagnosis [17]. Most children show improvement with broad-spectrum empirical coverage. However, in case of inadequate response to empirical therapy and clinical deterioration, more invasive procedures are recommended. Measurement of serum biomarkers, such as CRP, PCT or presepsin are proven to be very useful in earlier detection of patients that are unresponsive and therefore candidates to these procedures. The latter, presepsin, is a new and promising biomarker as there are recent studies supporting its superiority over CRP and PCT in prediction of bacterial infections in this group of patients [18,19]. Invasive procedures assist in the accurate identification of the causative agent that will help in the verification of its susceptibility to antimicrobial drugs in order to adjust the management strategy with the addition or discontinuation of certain pharmacological agents. Those valuable results are frequently obtained through BAL, CT-guided fine-needle aspiration or open lung biopsy (OLB) [20,21,22,23].

Recently, an increasing number of studies have shown the superior safety and effectiveness of BAL over the other methods with only few reported complications such as transient hypoxia and pulmonary hemorrhage [8,21,24]. More specifically, Chellapandian et al. in 2015 systematically reviewed 53 studies on the use of BAL or lung biopsy for the evaluation of pulmonary lesions in both adult and pediatric patients with cancer or post hematopoietic stem cell transplantation. Their meta-analysis resulted in an overall significant superiority of BAL in the diagnosis of infections. Further, they performed a stratified analysis of pediatric patients regarding the diagnostic potential of these procedures and their complication risk; although there was no statistically significant difference in determining an infectious diagnosis, BAL had lower complication risk when compared to biopsy (0.08 v 0.37, *p* = 0.001). Thus, BAL may be the method of choice when infection is suspected [25].

When the aforementioned procedures are unfeasible or unsuccessful, fluorine-18 fluorodeoxyglucose positron-emission tomography combined with CT (18F-FDG-PET/CT) imaging may be helpful early in the diagnostic algorithm, as shown in a prospective study of 48 adult patients with FN and hematological malignancy [26]. However, a clear recommendation for its routine use in pediatric neutropenic patients cannot be given due to lack of relevant studies.

### 2.4. Identified Causes

Pneumonia diagnosed during the induction therapy for acute leukemia is due to bacteria, viruses and fungi that can be found in Table 1 [27].

The impact of viruses in febrile neutropenia was recognized in the past decade. Children with chemotherapy-induced FN have a large number of samples positive for respiratory viruses (RV) [9,28,29,30,31]. RV are generally transmitted by large respiratory droplets, most frequently in seasonal epidemiology. *Rhinovirus (RhV)*, *Respiratory Syncytial Virus (RSV)*, *Influenza A*, *Parainfluenza (PIV)*, *Human Bocavirus (HBoV)* and *Human Metapneumovirus (HMOV)* are the commonly responsible viral agents. Moreover, *Cytomegalovirus (CMV)* can cause pneumonia (interstitial pneumonia or mixed) in patients undergoing bone marrow transplantation, while *Human Herpesvirus* (*HHV-6/7/8)* is responsible for interstitial pneumonia. In some patients, more than one virus can be identified, while other microbial agents coexist in one out of three cases.

The annual influenza vaccination is well established so as to lower the mortality among children with blood cancer receiving chemotherapy [32]. In a recent case control study, Meena et al. studied RV detection in nasopharyngeal swabs of 81 FN episodes with signs of acute respiratory infections (ARI) compared with those of 37 FN episodes without ARI. The authors reported that overall prevalence of RV infection was 76.5% in case group which differed significantly from the control group. The most commonly detected virus was *RhV* (40%; 25/62) followed by *RSV* seen in 17% of cases and *influenza A* and enteroviruses (seen in 14% of patients each) [33]. Similarly, high *RhV* prevalence among neutropenic children with FN has been reported by Barreiro et al. and Torres et al. [14,17]. *RhV*, *RSV*, *Adenovirus*, *Influenza*, *Parainfluenza* and *HMOV* can be detected with multiplex PCR. However, the clinical significance of a positive PCR result for *RhV* has been questionable, as *RhV* is also frequently isolated from healthy asymptomatic children [17,34]. Thus, the detection of a respiratory virus in children with FN cannot lead to safe conclusions regarding the causative agent, especially in children with signs of severe infection [17]. Additional controlled studies are needed to determine the exact role of RV in these patients.

What is more, regarding to the current pandemic of *SARS-CoV-2*, the causative agent of Corona Virus Disease 2019 (COVID-19), several studies have reported a mild, mainly asymptomatic course of the disease in pediatric patients [35,36]. However, there are no adequate epidemiologic data about the way chemotherapy-induced immunosuppression affects the susceptibility of children with blood cancer. Lu et al. reported one case out of 171 children tested positive for *SARS CoV-2* infection, who was under chemotherapy treatment for leukemia and required admission to Intensive Care Unit (ICU) with no other major events. General recommendations concerning the management of pediatric cancer patients during the pandemic have already been published [37], but further studies are required for more conclusive results. 

Neutropenia is the major predisposing factor for bacterial infections. It has been shown that Gram-negative bacteria are the most commonly isolated organisms in neutropenic pneumonia [7,38].

According to data presented by Armenian et al., 113 pediatric cancer patients with pulmonary infiltrates unresponsive to empirical antibacterial treatment for FN underwent an invasive diagnostic procedure such as BAL, CT biopsy or OLB, identifying 63 organisms in 56 pulmonary infiltrates out of which 43% were fungi, 36% viruses and 21% bacteria. More than half of the fungal infections were due to *Aspergillus* spp. (24% of overall infections) while less common fungal pathogens included *Candida* spp. (10%), *Pneumocystis jirovecii* (8%) and *Fusarium* spp. (3%). The most common identified bacteria were *Pseudomonas* spp. (5%), *Staphylococcus aureus* (5%), *Nocardia* spp. (3%) and *Group D enterococcus* (3%) [39].

While the frequency of *Aspergillus* spp. isolation in lung samples has been rising according to multiple studies [25,40,41], the incidence of *Pneumocystis jirovecii* pneumonia has dropped significantly since effective chemoprophylaxis with co-trimoxazole was established in children undergoing chemotherapy for hematologic malignancies [42,43].

### 2.5. Treatment and Prognosis

Children with FN are systematically categorized as high- and low-risk for serious infectious complications according to specific criteria that take into account the initial patient presentation, ANC and estimated duration of the neutropenic episode, and the type of the underlying malignancy among others [10,12]. Regarding the risk stratification, the patient status (age, degree and duration of neutropenia, colonizing microbial flora, CVC), the therapeutic approach (dose intense and time of chemotherapy) and the characteristics of the episode (fever, blood pressure, CRP) are of major concern. Even if we take under consideration all these factors, there is no consensus for risk stratification.

Prompt initiation of broad-spectrum antibacterial agents is the keystone that determines a favorable outcome [44]. Patients who receive antibiotics for fever and neutropenia in less than 60 min from the first fever have decreased ICU needs [45].

According to current guidelines [10], high-risk children should be treated in an inpatient setting with the appropriate intravenous regimen. Empiric antibiotic therapy coverage must cover organisms isolated at individual institutions, including *Pseudomonas* spp. Between widely used empiric regimens is either monotherapy with fourth generation antipseudomonal cephalosporin, carbapenem (imipenem/cilastatin, meropenem), piperacillin-tazobactam or dual therapy (antipseudomonal β-lactam plus an aminoglycoside). Multiple meta-analyses have shown that monotherapy is not inferior to dual therapy [12].

In case of documented pneumonia on imaging, aminoglycoside and/or glycopeptides (vancomycin, teicoplanin) are part of the first line empirical treatment. Glycopeptides are added whenever methicillin-resistant *Staphylococcus aureus* infection is suspected. Once-a-day dosing of aminoglycosides is thought to be more effective and less nephrotoxic when compared to twice or thrice daily.

Unresponsive fever of more than 4 days should be addressed by the addition of antifungal agents, such as micafungin, caspofungin or liposomal amphotericin B (L-Amb) on the curative regimen. In general, antifungal agents act at the fungal cell membrane (polyens), ergosterol synthesis (fluconazole, itraconazole, voriconazole, posaconazole, isavuconazole) or the cell wall (caspofungin, micafungin, anidulafungin).

Documented viral infections are managed with antiviral treatment, while the prophylaxis against them includes vaccination, chemoprophylaxis and immunoprophylaxis. Specifically, with regard to the seasonal flu in the time of epidemic maximum spread, in the presence of respiratory symptoms or fever, it is advisable to start oseltamivir at a dose of 75–100 mg orally twice daily for 5–10 days or until negative virology results. For *adenovirus*, the treatment is cidofovir intravenously 5 mg/kg/week for 3 weeks and then continuation with probeneside every 2 weeks. For *RSV*, orally or intravenously (IV) ribavirin at a dose of 10 mg/kg once and then 20 mg/kg/d, and immunoglobulin (IVIG) or IV palivizumab. Palivizumab is given once at a dose of 15 mg/kg [46,47]. For *Parainfluenza* and *Human Metapneumonovirus*, ribavirin has been recommended. For Corona viruses, the management is mainly supportive, while ribavirin should be administered especially in case of *SARS-CoV* infection. For *Parvovirus B19*, use of IVIG has been recommended. For *Rhinovirus*, *Bocavirus*, *WU* and *KI* viruses, there is no treatment available, except for supportive care [48].

Whenever it happens during the treatment, identification of the causal agent modifies the therapeutic management based on culture and susceptibility test results. The total duration of therapy is usually individualized and highly depends on the clinical and bone marrow recovery [10,12]. Lastly, it is not fully clarified whether the use of haematopoietic growth factors, such as granulocyte colony stimulating factors (G-CSF), are beneficial or not for patients with high risk for prolonged neutropenia [49]. G-CSF is recommended when the possibility of a neutropenic fever episode is over 20% according to the NICE guidelines on neutropenic sepsis of 2012. It has been found to improve survival and decrease the rate of bacterial and fungal infections [50].

Overall, the reported mortality rate of pulmonary infectious diseases in this population is up to 20% with higher rates seen in cases unresponsive to initial therapy and those who required mechanical ventilation during their management [6,51,52].

## 3. Conclusions

Pulmonary infections of pediatric patients undergoing chemotherapy require careful and thorough evaluation, as they are associated with increased mortality rate that can reach 20%. Although respiratory viruses are the most frequently identified pathogens, their clinical importance is questionable. Fungi are the most commonly detected causative agents in children unresponsive to empirical antibacterial therapy. Identification of the causative organism in these immunocompromised patients is crucial, in terms of applying the best management and treatment plan. In light of the lack of pathogen identification and unresponsiveness to antibacterial medications, a more invasive testing should be pursued in order to isolate the responsive pathogen. Finally, treatment management should be based on the severity of the disease, while having a low threshold to initiate a more invasive approach in patients unresponsive to initial treatment and those who require mechanical ventilation in ICU.

## Figures and Tables

**Table 1:** Commonly identified respiratory pathogens in children with chemotherapy-induced FN.

Viruses	Bacteria	Fungi
*Rhinovirus (RhV)* *Respiratory Syncytial Virus (RSV)* *Influenza A* *Parainfluenza (PIV)* *Human Bocavirus (HBoV)* *Human Metapneumovirus (HMOV)* *Cytomegalovirus (CMV)* *Human Herpesvirus (HHV-6/7/8)* *Adenovirus* *Coronavirus* (i.e., *SARS-CoV-2*)	*Pseudomonas* spp. *Klebsiella* spp. *Escherichia coli* *Streptococcus pneumoniae Staphylococcus aureus* *Nocardia* spp. *Group D enterococcus* *Mycoplasma* spp. *Chlamydia trachomatis*	*Aspergillus* spp. *Candida* spp. *Fusarium* spp. *Pneumocystis jirovecii*

FN, Febrile Neutropenia; spp, species.
